# A novel hypoxia gene signature indicates prognosis and immune microenvironments characters in patients with hepatocellular carcinoma

**DOI:** 10.1111/jcmm.16249

**Published:** 2021-02-22

**Authors:** Qiangnu Zhang, Lijun Qiao, Juan Liao, Quan Liu, Pengyu Liu, Liping Liu

**Affiliations:** ^1^ Department of Hepatobiliary and Pancreas Surgery The Second Clinical Medical College, Jinan University (Shenzhen People’s Hospital) Shenzhen Guangdong China; ^2^ Integrated Chinese and Western Medicine Postdoctoral Research Station Jinan University Guangzhou China; ^3^ Department of Gastroenterology West China School of Public Health and West China Fourth Hospital Sichuan University Chengdu China; ^4^ Department of Gastroenterology and Hepatology Erasmus MC‐University Medical Center Rotterdam NY USA; ^5^ Department of Hepatobiliary and Pancreas Surgery, the First Affiliated Hospital, Southern University of Science and Technology Shenzhen Guangdong China

**Keywords:** gene signature, HCC, hypoxia, microenvironment, prognosis

## Abstract

Due to the lack of a suitable gene signature, it is difficult to assess the hypoxic exposure of HCC tissues. The clinical value of assessing hypoxia in HCC is short of tissue‐level evidence. We tried to establish a robust and HCC‐suitable hypoxia signature using microarray analysis and a robust rank aggregation algorithm. Based on the hypoxia signature, we obtained a hypoxia‐associated HCC subtypes system using unsupervised hierarchical clustering and a hypoxia score system was provided using gene set variation analysis. A novel signature containing 21 stable hypoxia‐related genes was constructed to effectively indicate the exposure of hypoxia in HCC tissues. The signature was validated by qRT‐PCR and compared with other published hypoxia signatures in multiple large‐size HCC cohorts. The subtype of HCC derived from this signature had different prognosis and other clinical characteristics. The hypoxia score obtained from the signature could be used to indicate clinical characteristics and predict prognoses of HCC patients. Moreover, we reveal a landscape of immune microenvironments in patients with different hypoxia score. In conclusion, we identified a novel HCC‐suitable 21‐gene hypoxia signature that could be used to estimate the hypoxia exposure in HCC tissues and indicated prognosis and a series of important clinical features in HCCs. It may enable the development of personalized counselling or treatment strategies for HCC patients with different levels of hypoxia exposure.

## 1 INTRODUCTION

In 2018, there were 841 080 new cases of liver cancer worldwide, ranking sixth in the global incidence of all cancers. In the same year, liver cancer caused 781 631 deaths worldwide, ranking as the fourth leading cause of cancer death in the world.^1^ Hepatocellular carcinoma (HCC) accounts for 85%‐95% of primary liver cancers.^2^ Due to the insidious onset and inadequate early diagnosis measures, 80% of patients are already in the middle and late stages of disease at the time of diagnosis, thus missing the optimal time for surgery.^3^ In patients with advanced HCC, the mortality rate is as high as 80%, the median survival is <1 year, and the 5‐year survival rate is <20%.^4^ Although the development of targeted therapy and immunotherapy for the treatment of HCC has brought new hope to patients with advanced HCC, the overall efficacy of these therapeutic methods remains dismal.^5^

The initiation and progression of HCC involve interactions with the hypoxia microenvironment which regulating invasiveness, angiogenesis, stemness, metabolic reprogramming, immune response and resistance to radiochemotherapy.^6^, ^7^ Therefore, revealing the hypoxia exposure in HCC tissues will be conducive to the early diagnosis, predicting prognosis, finding treatment targets and to establishing personalized treatment.^8^ At present, there is no convenient and quick way to accurately assess the degree of hypoxia in HCC tissue. Because hypoxia exposure can cause changes in gene expression levels, researchers have begun to use hypoxia‐related gene signatures to reflect the hypoxia of tumour tissues. A series of excellent gene signatures that reflect the hypoxia level in tumour tissues has been developed in the last decade. For instance, Buffa’s hypoxia signature,^9^ Eustace's hypoxia signature^10^ and Ragnum’s hypoxia signature^11^ are common signatures to estimate the tissue hypoxia in Pan‐cancer studies. However, until now HCC‐suitable hypoxia signature was only reported by few studies. Malenstein et al have developed a 7‐gene set associated with hypoxia exposure in HCC that can be used to indicate the prognosis of patients.^12^ But the 7‐gene signature was only collected from 72 hours hypoxia‐treated HEPG2 cells and validated in 4 data sets. To obtain a more robust HCC‐specific hypoxia signature, more HCC cell lines, long‐term intermittent hypoxia model and comprehensive validation based on multiple large‐size cohorts should be considered. In the present study, we collected and integrated hypoxia‐induced mRNA profiles across three microarray data sets including three different hypoxia‐treated HCC cells. We selected and established a signature based on a panel of 21 genes that were consistently responsive to hypoxia treatment under acute, chronic and intermittent hypoxia conditions. The robustness of this novel 21‐gene hypoxia signature was validated in another 3 microarray data sets from HCC cell lines and 11 HCC patients’ data sets. Then, the association of the signature with clinical characteristics, prognoses and immune microenvironments of HCC patients was analysed. We believe that the proposed hypoxia gene signature will provide useful information for the diagnosis and treatment of HCC.

## 2 METHODS

### 2.1 Cell culture and hypoxia treatment

HCC cell lines including HUH7, SNU‐182 and HLF cells were used in the present study. HUH7 and HLF cells from the Japanese Cancer Research Resources Bank. SNU‐182 cells were obtained from American Type Culture Collection. HUH7 and HLF cells were cultured in Dulbecco's Modified Eagle Medium (DMEM, high glucose). SNU‐182 were cultured in RPMI 1640 Medium. All medium was supplemented with 10% foetal calf serum (FCS) and 1% Penicillin (100 IU/m)‐Streptomycin (100 g/mL) solution. Cells were maintained in an incubator with 37°C, 5%CO_2_ and 95% relative saturation of humidity. For hypoxia treatment, cells were cultured under an atmosphere of 1% O_2/_5% CO_2_/94% N_2_ for 24 hours.

### 2.2 RNA extraction and microarray

Hypoxia‐treated HUH7, SNU‐182 and HLF cells were collected. Total RNA was extracted from treated cells using TRIzol Reagent following the protocol from the manufacturer's instructions. The concentration and quality of total RNA were measured using NanoDrop microvolume spectrometer. The RNA samples with high quality were labelled and hybridized on Agilent Whole human genome chip (4 × 44 K). Differentially expressed mRNAs were extracted using R software (version 3.6.1) with Limma package.

### 2.3 Real‐time quantitative reverse transcription PCR

Total RNA was quantified and reverse transcribed into complementary DNA (cDNA) using PrimeScript™ RT Reagent Kit (Takara Bio, Inc). Then, the cDNA samples were analysed using the SYBR^®^ Premix Ex Taq™ II Kit (Takara Bio, Inc).

### 2.4 Generation of a novel hypoxia gene signature and calculation of hypoxia score

Our novel hypoxia gene signature was built using robust rank aggregation (RRA)algorithm^13^ from the microarray data of hypoxia‐treated HUH7, SNU‐182 and HLF cells. Up‐regulated (fold change > 1.5) mRNAs with *P* < .05 were selected from the RRA output. 21 mRNAs were included after qRT‐PCR validation under acute, chronic and intermittent hypoxia conditions. To estimate the hypoxia exposure, the hypoxia score was calculated using gene set variation analysis (GSVA).^14^ We also compared our 21‐gene hypoxia signature with seven other published hypoxia gene signatures including Buffa’s signature,^9^ Eustace's signature,^10^ Ragnum’s signature,^11^ Sorensen's signature,^15^ winter’s signature^16^ and Malenstein’s signature.^12^ Hypoxia scores based on these seven signatures were also calculated by GSVA. The gene lists of 21‐gene signature were shown in Table S1.

### 2.5 Public data sets

We retrieved three independent mRNA microarray data sets (GSE18494, GSE55214 and GSE57613) based on hypoxia‐treated HCC cells from the Gene Expression Omnibus (GEO, https://www.ncbi.nlm.nih.gov/geo/). data sets and available clinical information of ten HCC cohorts including GSE14520 (n = 224), GSE22058 (n = 100), GSE25097 (n = 268), GSE36376 (n = 240), GSE45436 (n = 48), GSE64041 (n = 60), GSE76297 (n = 32), GSE76427 (n = 115), GSE10141 (n = 80), GSE9843 (n = 91) and GSE6764 (n = 75) were downloaded from GEO. All gene symbols in GEO data sets were converted to the latest (HUGO Gene Nomenclature Committee) HGNC Symbols. Level 3 mRNA expression data of the Cancer Genome Atlas Liver Hepatocellular Carcinoma (TCGA‐LIHC, n = 356) were downloaded from the TCGA data portal (https://portal.gdc.cancer.gov/).

### 2.6 Biological process and pathway enrichment assay

The biological process and pathway enrichment assay of candidate genes were performed using online tools provided by Metascape (http://metascape.org/gp/index.html). The enrichment analysis has been carried out with the following ontology sources: KEGG Pathway, GO Biological Processes, Reactome Gene Sets, Canonical Pathways and CORUM. All genes in the genome have been used as an enrichment background. To establish the network based on the relationships between the terms, a subset of enriched terms with a similarity > 0.3 were connected by edges. 20 clusters were obtained, and the terms with the best *P*‐values were selected (for more details see the website of Metascape). The network is visualized using Cytoscape (version 3.7.2).

### 2.7 Protein‐protein interaction enrichment analysis

We used online tools provided by Metascape to perform protein‐protein interaction enrichment analysis for the production of candidate genes. According to data from BioGrid, InWeb_IM and OmniPath, a resultant network contains the subset of proteins that form physical interactions with at least one other member was built using Molecular Complex Detection (MCODE) algorithm.^17^ Then, Pathway and process enrichment analysis has been applied to each MCODE component (for more details see the website of Metascape). The network is visualized using Cytoscape (version 3.7.2).

### 2.8 Estimation for immune microenvironment

The presence of infiltrating stromal and immune cells in tumour tissues was predicted using ESTIMATE algorithm (Estimation of Stromal and Immune cells in Malignant Tumor tissues).^18^ ESTIMATE algorithm provided stromal score (that captures the presence of stroma) and immune score (that represents the infiltration of immune cells) based on mRNA expression of tumour tissues. The relative levels of distinct immune cell types were estimated using CIBERSORT tools (https://cibersort.stanford.edu) with LM22 files as reference.

### 2.9 Statistical analysis

Statistical analyses were performed using R software (version 3.6.1) with relevant packages. In brief, the differential expressed mRNAs were extracted from microarray data sets using Limma package. The differential expressed mRNAs, microRNA and lncRNA of TCGA‐LIHC were identified using Linnorm packages. The difference between the two groups was compared using an independent *t* test or Wilcox test. Adjusted *P*‐value was obtained using FDR (False Discovery Rate) method. Coefficients were calculated using Pearson or Spearman correlation analysis. A chi‐squared test was used to determine the significant difference between the frequencies. Survival analysis was performed using Univariate Cox/multivariate analysis hazard analysis or Kaplan‐Meier survival estimate using a survival package. The forest‐plot R package was employed to visualize the hazard rate obtained from survival analysis. The Kaplan‐Meier survival curves were created using survminer package with Log‐rank test. A prognostic model based on hypoxia score was established using Lasso (least absolute shrinkage and selection operator)‐cox Regression method. The model was visualized and validated using Hdnom package. In the present study, statistical significance was set at a probability value of *P* < .05.

## 3 RESULTS

### 3.1 Identification for a novel HCC‐suitable hypoxia signature that includes 21 genes

**FIGURE 1:**
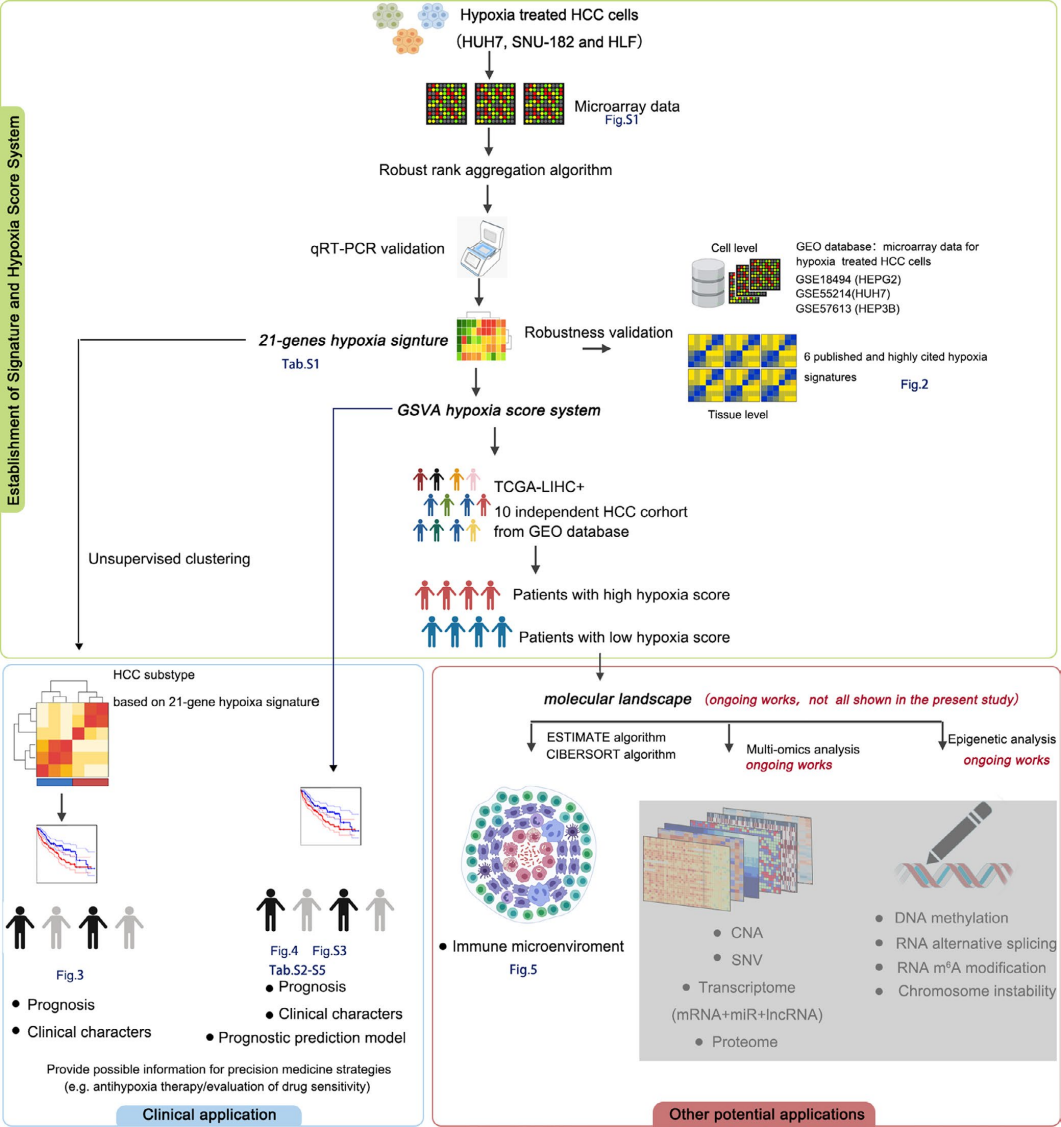
The overall design and content of this study

**FIGURE 2:**
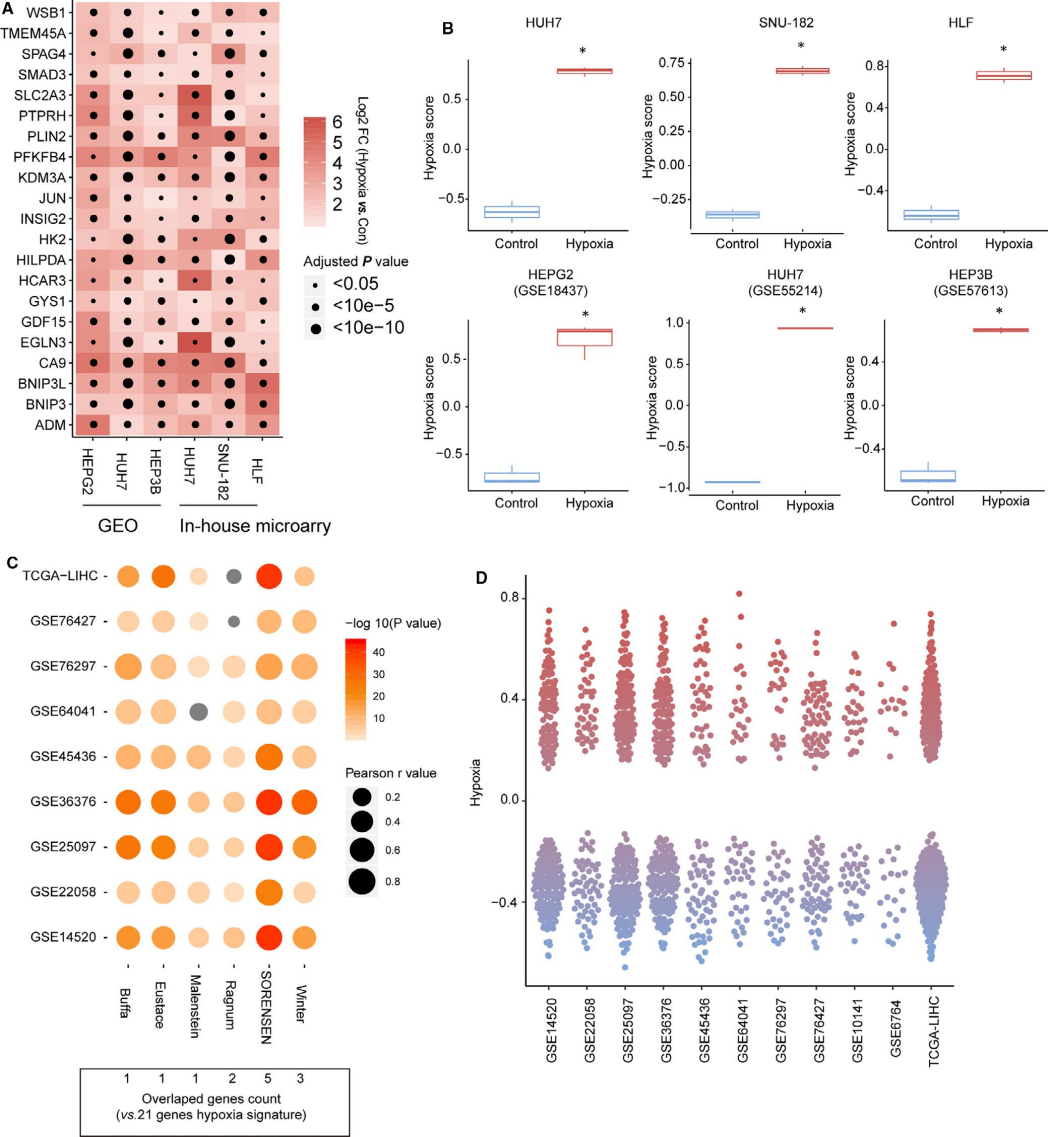
The 21‐gene hypoxia signature indicated hypoxia exposure in hepatocellular carcinoma (HCC) cells. A, Expression changes in 21 genes in 6 HCC cells after hypoxia exposure. B, Hypoxia scores calculated based on the 21‐gene hypoxia signature using gene set variation analysis (GSVA) significantly increased in hypoxia‐treated HCC cells. C, Correlations between the hypoxia scores calculated based on the 21‐gene hypoxia signature and the hypoxia score calculated based on other published hypoxia signatures in the cancer tissues of 9 HCC cohorts. D, The distribution of hypoxia scores was calculated based on the 21‐gene hypoxia signature in the cancer tissues of 11 HCC cohorts. *Compared with the control group, *P* < .05

The overall design of this study is shown in Figure 1. First, through microarray analysis, differentially expressed genes with fold changes (FC) satisfying log_2_FC > 0.58 or log_2_FC < −0.58 and *P* < .05 in HUH7, SNU‐182, and HLF cells were obtained after 24 hours of hypoxia exposure. The profiles and functional analysis of these differentially expressed genes in microarray analysis are shown in Figure S1. The results of functional analysis strongly indicated the differentially expressed genes were hypoxia‐related. Next, we started to construct a new hypoxia signature based on the data obtained according to a design principle that involved using a minimum number of genes to collectively indicate the hypoxia‐related phenotype of interest. The differentially expressed genes of 3 hypoxia‐treated HCC cell lines were screened and integrated using the robust rank aggregation (RRA) algorithm. The integrated results were confirmed by qRT‐PCR (Figure S2). We found 21 genes were stably induced by acute hypoxia (24 hours), chronic hypoxia (72 hours) and intermittent hypoxia (24 hours hypoxia/24 hours reoxygenation, 1 week). Hence, these 21 were selected to construct the hypoxia signature. The detailed information for these 21 genes is shown in Table S1. The expression of these 21 genes was significantly increased in HUH7, SNU‐182 and HLF cells after hypoxia exposure. We also found 3 hypoxia‐related microarray data sets in the Gene Expression Omnibus (GEO) database, including mRNA expression in hypoxia‐treated HEPG2 (GSE18494), HUH7 (GSE55214) and HEP3B (GSE57613) cells. In the 3 data sets, the 21 genes that were selected to construct the hypoxia signature were also significantly up‐regulated after hypoxia exposure (Figure 2A). Therefore, these 21 genes are hypoxia‐responsive in HCC cells. Next, gene set variation analysis (GSVA) was used to calculate the hypoxia score. Compared with the control group, hypoxia‐treated HCC cells had significantly higher hypoxia scores (Figure 2B). Therefore, the hypoxia scores calculated based on the 21‐gene hypoxia signature can reflect the hypoxic condition in HCC cells. To further prove the robustness of the 21‐gene hypoxia signature in the assessment of hypoxia, 6 hypoxia signatures that have been reported in highly cited articles were selected to calculate hypoxia scores: Buffa's signature (15 genes), Eustace's signature (26 genes), Ragnum's signature (32 genes), Sorensen's signature (27 signature), Winter's signature (99 genes) and Malenstein's signature (7 genes). The first 5 have been proven to be excellent performers in recent pan‐cancer comprehensive studies assessing the robustness of different hypoxia signatures.^19^, ^20^ Malenstein's signature is a liver‐specific hypoxia signature associated with HCC prognosis.^12^ There were significantly positive correlations between hypoxia scores calculated based on our 21‐gene hypoxia signature and those calculated based on the other 6 signatures for cancer tissues from 9 HCC data sets, including The Cancer Genome Atlas‐Liver Hepatocellular Carcinoma (TCGA‐LIHC, n = 365), GSE14520 (n = 224), GSE22058 (n = 100), GSE25097 (n = 268), GSE36376 (n = 240), GSE45436 (n = 48), GSE64041 (n = 60), GSE76297 (n = 32) and GSE76427 (n = 115) (Figure 2C). Cell‐ and tissue‐level evidence suggests that our 21‐gene signature is robust for the assessment of hypoxia. The hypoxia scores for liver cancer tissues were clustered in 2 groups (Figure 2D), positive and negative, suggesting that the degrees of exposure of liver cancer tissues to hypoxia in different patients are different.

### 3.2 HCC subtypes obtained based on the 21‐gene hypoxia signature have different clinical characteristics and prognoses

**FIGURE 3:**
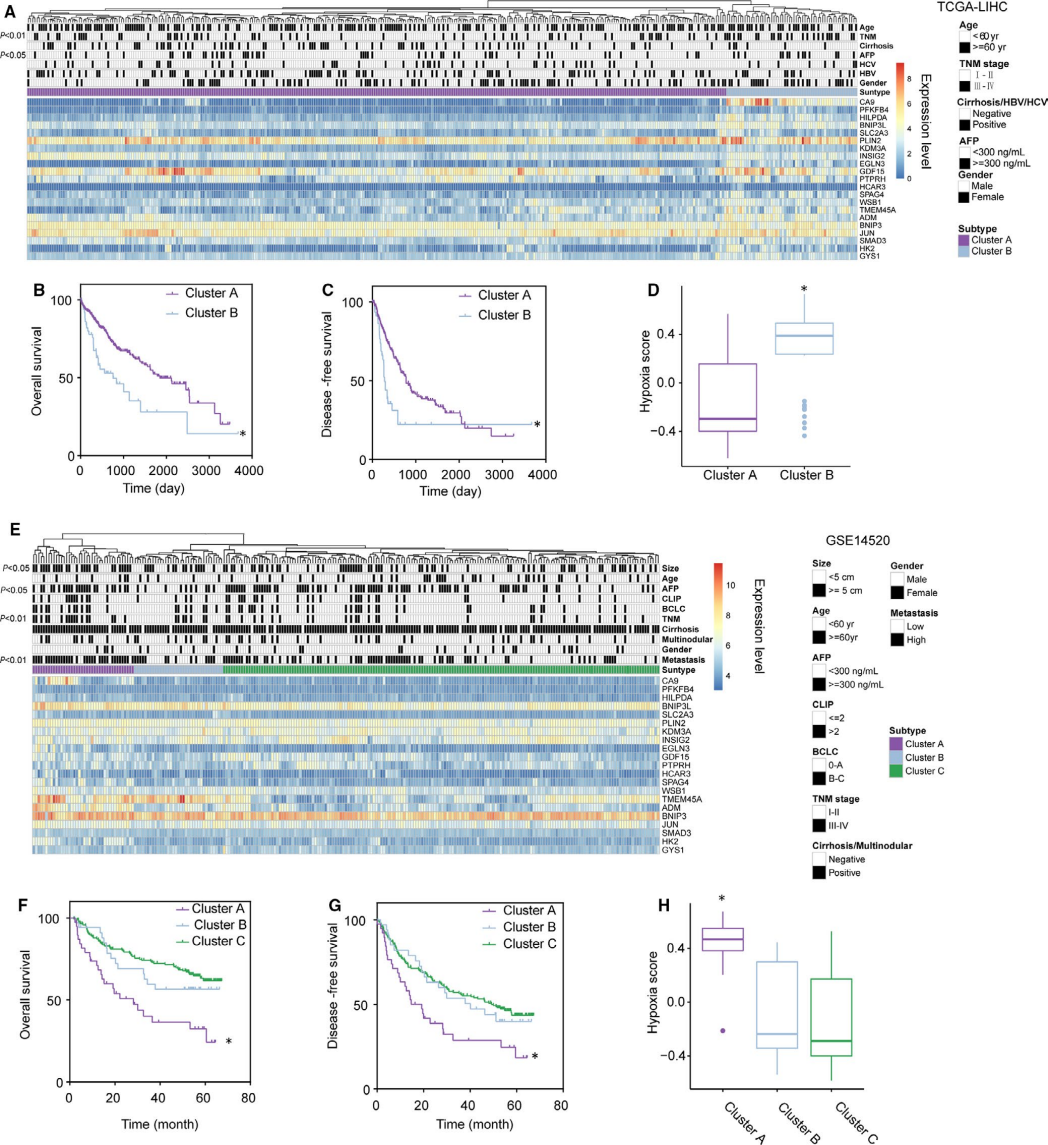
Hepatocellular carcinoma (HCC) subtypes classified using the 21‐gene hypoxia signature are correlated with clinical characteristics and patient prognoses. A, Unsupervised clustering based on the 21‐gene hypoxia signature grouped patients in TCGA‐LIHC into 2 subtypes. There were significant differences in the tumour‐node‐metastasis (TNM) stage and alfa‐fetoprotein (AFP) levels between the 2 subtypes. B and C, Differences in OS and DFS between the 2 subtypes in TCGA‐LIHC. D, Difference in hypoxia scores between the 2 subtypes in TCGA‐LIHC. E, Unsupervised clustering based on the 21‐gene hypoxia signature grouped patients in GSE14520 into 3 subtypes. There were differences in tumour size, AFP levels, TNM stage, and invasiveness between cluster A and the other clusters. F and G, Differences in OS and DFS between the 3 subtypes in GSE14520. H, Differences in hypoxia scores between the 3 subtypes in GSE14520. *Compared with other clusters, *P* < .05

Unsupervised hierarchical clustering analysis was used to group patients (n = 374) in the TCGA‐LIHC data set based on the 21‐gene signature. All patients were grouped into 2 subtypes based on the 21‐gene signature (Figure 3A): LIHC‐cluster A (n = 315) and LIHC‐cluster B (n = 59). The proportion of patients with stage III‐IV HCC, according to the tumour‐node‐metastasis (TNM) staging system, in LIHC‐cluster B was higher (*χ* ^2^ = 11.35, *P* < .01) than that in LIHC‐cluster A, and the alfa‐fetoprotein (AFP) level in LIHC‐cluster B was higher than that in LIHC‐cluster A (*χ* ^2^ = 4.44, *P* < .05). The overall survival (OS) rate (Figure 3B, hazard ratio (HR) = 2.15, log‐rank *P* < .01) and disease‐free survival (DFS) (Figure 3C, HR = 2.17, log‐rank *P* < .01) of patients in LIHC‐cluster B were significantly lower than those of patients in LIHC‐cluster A. The hypoxia scores of patients in LIHC‐cluster B were significantly higher than those of patients in LIHC‐cluster A (Figure 3D). Similar unsupervised hierarchical clustering analysis grouped the patients in GSE14520 into 3 subtypes (Figure 3E): GSE14520‐cluster A (n = 41), GSE14520‐cluster B (n = 35) and GSE14520‐cluster C (n = 172). Compared with the other 2 subtypes, GSE14520‐cluster A had a significantly higher proportion of patients with high TNM stages (*χ* ^2^ = 15.07, *P* < .01) and higher AFP levels (*χ* ^2^ = 7.54, *P* < .05). In GSE14520‐cluster A, the number of cases with a tumour diameter >5 cm was also higher than that in the other 2 subtypes (*χ* ^2^ = 9.91, *P* = .01). Roessler et al used a metastasis gene signature to group GSE14520 patients into patients with high invasion risk and patients with low invasion risk.^21^ In GSE14520‐cluster A, the proportion of patients with high invasion risk was significantly higher than in the other 2 subgroups (*χ* ^2^ = 22.36, *P* < .01). As expected, the OS rate (Figure 3F, HR_A: B_ = 1.31, HR_A: C_ = 2.71, log‐rank *P* < .01) and DFS (Figure 3G, HR _A: B_ = 1.81, HR _A: C_ = 1.94, log‐rank *P* < .01) of patients in GSE14520‐cluster A were lower than those of patients in other subtypes. Patients with a poor prognosis in GSE14520 cluster A had high hypoxia scores (Figure 3H). The evidence suggests that the molecular classification of HCC based on the 21‐gene hypoxia signature can reflect different clinical features and prognoses.

### 3.3 Hypoxia scores calculated based on the 21‐gene hypoxia signature can be used as a prognostic marker that indicates HCC progression

**FIGURE 4:**
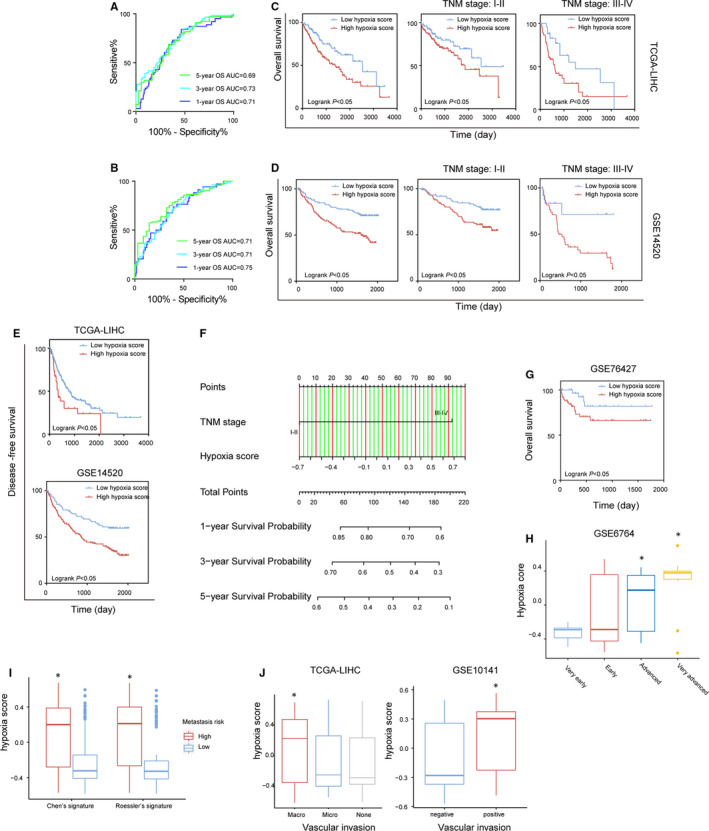
The hypoxia scores calculated based on the 21‐gene hypoxia signature can predict the prognoses of patients with hepatocellular carcinoma (HCC). A and B, The time‐dependent receiver operating characteristic (ROC) curves for overall survival (OS) of patients in TCGA‐LIHC and GSE14520 estimated by hypoxia scores. C and D, The differences in OS between patients with high hypoxia scores and low hypoxia scores in TCGA‐LIHC and GSE14520. After stratification of patients using TNM staging, the differences in OS between patients with high hypoxia scores and low hypoxia scores were compared again. The cut‐off was calculated using X‐tile software. E, The differences in DFS between the patients with high hypoxia scores and low hypoxia scores in TCGA‐LIHC and GSE14520. F, Nomogram of the LASSO‐cox regression model to predict 1‐, 3‐ or 5‐year OS in TCGA‐LIHC using hypoxia score and TNM stage. G, The difference in OS between patients with high hypoxia scores and low hypoxia scores in GSE76427. The cut‐off value was calculated using X‐tile software. H, The hypoxia scores for patients in GSE6764 increased with the progression of the clinical stage. I, The differences in hypoxia scores between HCC patients with different metastasis risk. J, Correlations between hypoxia scores and vascular invasion in patients in TCGA‐LIHC and GSE10141. *Compared with other groups, *P* < .05

The results of the univariate and multivariate Cox regression analyses (Tables S2 and S3) indicate that a high hypoxia score is an independent risk factor for OS in HCC patients in the TCGA‐LIHC and GSE14520 data sets (HR = 1.82 in TCGA‐LIHC and HR = 2.71 in GESE14520, all *P* < .05). In TCGA‐LIHC and GSE14520, the hypoxia score was used to evaluate the receiver operating characteristic (ROC) curves for 1‐, 3‐ and 5‐year survival and the corresponding areas under the curves (AUCs) (Figure 4A,B). The optimal cut‐off value was calculated using X‐tile software,^22^ and the patients were divided into a high hypoxia score group and a low hypoxia score group. The OS rate for patients in the high hypoxia score group was lower than that for patients in the hypoxia score group. After stratification of patients using TNM staging, a high hypoxia score still implied poor OS (Figure 4C,D). In the TCGA‐LIHC (n = 365) and GSE14520 (n = 224) data sets, patients in the high hypoxia score group had shorter DFS than did patients in the low hypoxia score group (Figure 4E). The TCGA‐LIHC data set was used as the training set, and TNM stages and hypoxia scores were used as factors to construct the least absolute shrinkage and selection operator (LASSO)‐Cox regression model (c‐index = 0.73) for predicting the OS of patients, and a predictive nomogram was established accordingly (Figure 4F). The effect of the model was verified in the GSE14520 data set (Figure S3). In another HCC data set, GSE76427 (n = 115), patients with a high hypoxia score also had a low OS rate (Figure 4G). Therefore, the hypoxia score calculated based on the 21‐gene signature has the potential to be a prognostic marker for HCC patients. Compared with the low hypoxia score group in the TCGA‐LIHC data set, the high hypoxia score group (≥median hypoxia score) had a higher number of cases with late TNM stages. In the GSE14520 data set, patients in the high hypoxia score and low hypoxia score groups differed significantly in AFP level, degree of cirrhosis, tumour size, TNM stage, Barcelona Clinic Liver Cancer (BCLC) stage, Cancer of the Liver Italian Program (CLIP) stage and multinodular status, suggesting that a high hypoxia score is an unfavourable factor for HCC patients and can indicate progression (Tables S4 and S5). In the GSE6764 data set (n = 75), the hypoxia score in HCC tissues gradually increased from very early to very advanced HCC (Figure 4H). Metastasis is an important step in the progression of HCC. Roessler and Chen independently used 2 different signatures to group patients in the GSE14520 data set as having a high metastasis risk or a low metastasis risk.^21^, ^23^ We found that patients with a high metastasis risk had higher hypoxia scores, suggesting that hypoxia scores can indicate the risk of metastasis in HCC patients (Figure 4I). The data from the TCGA‐LIHC and GSE10141 data sets (n = 80) suggest that high hypoxia scores can indicate vascular invasion (Figure 4J).

### 3.4 Immune cell infiltration and hot immune checkpoints changes in HCC patients with different hypoxia scores

**FIGURE 5:**
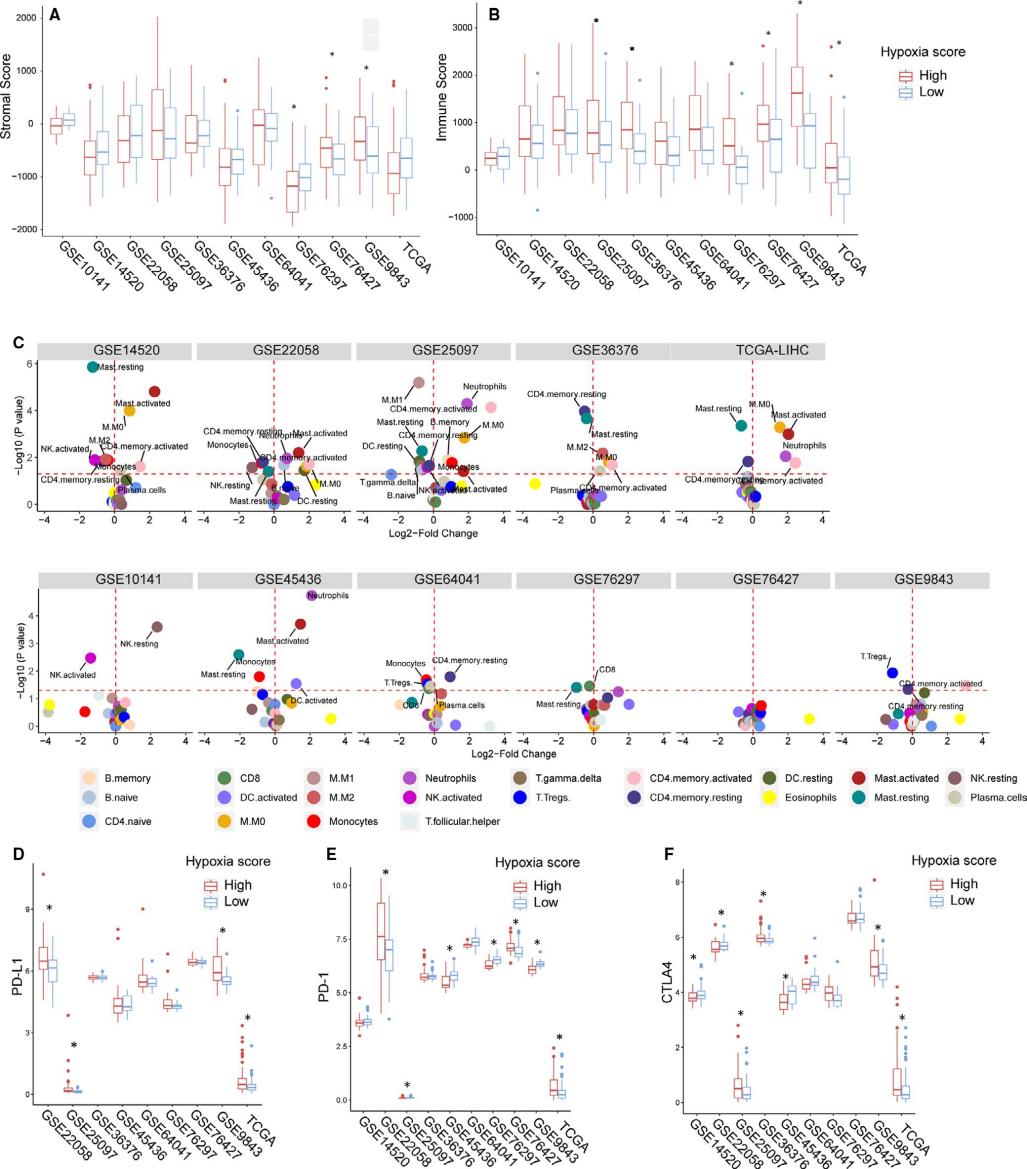
Differences in immune microenvironments between hepatocellular carcinoma (HCC) patients with high hypoxia scores and low hypoxia scores. A and B, Differences in stromal scores and immune scores (obtained using the ESTIMATE algorithm based on mRNA data) for cancer tissues between HCC patients with high hypoxia scores and low hypoxia scores in 11 data sets. C, Differences in the types of infiltrating immune cells (obtained using the CIBERSORT algorithm based on mRNA data) in cancer tissues between HCC patients with high hypoxia scores and low hypoxia scores in 11 data sets. E and F, Differences in the expression levels of programmed death‐1 (PD‐1), programmed death‐ligand 1 (PD‐L1) and cytotoxic T‐lymphocyte antigen 4 (CTLA4) between HCC patients with high hypoxia scores and low hypoxia scores in 11 data sets. *Compared with the low hypoxia score group, *P* < .05

The ESTIMATE algorithm was used to calculate the stromal and immune scores for patients in 11 HCC data sets. These 2 scores represent the infiltration of stromal cells and immune cells in tumour tissues. In GSE76297 (n = 32), GSE76427 and GSE9843, the stromal scores for tumour tissues were significantly higher in the high hypoxia score group than in the low hypoxia score group, whereas in other data sets, the stromal scores for tumour tissues were not significantly different between the 2 groups (Figure 5A). In GSE76297, GSE7642, GSE9843, GSE25097, GSE36376 and TCGA‐LIHC, the immune scores for tissues with high hypoxia scores were significantly increased (Figure 5B), and in other data sets (except for GSE10141), the immune scores for tissues with high hypoxia scores were also increased but not significantly. Almost all data sets showed that immune scores were higher in patients with high hypoxia scores than in patients with low hypoxia scores. The differences in immune scores between the 2 groups were significant in the GSE76297, GSE9843, GSE25097, GSE36376 and TCGA‐LIHC data sets, suggesting that the degree of immune cell infiltration was higher in patients with high hypoxia scores than in patients with low hypoxia scores. We also used the CIBERSORT algorithm. The distribution of 22 types of immune infiltrating cells in HCC tissues in 11 data sets was evaluated. Figure 5C shows the different types of infiltrating immune cells in the high hypoxia score group and the low hypoxia score group. Here, we need to emphasize some results with high consistency in multiple data sets. In GSE14520, GSE22058, GSE25097, GSE36376, GSE9843 and TCGA‐LIHC, a significant reduction in the proportion of infiltrating CD4 memory resting cells was accompanied by a significant increase in the proportion of CD4 memory activated cells in tissues with high hypoxia scores. In GSE14520, GSE22058, GSE25097, GSE45436 and TCGA‐LIHC, a significant reduction in the proportion of infiltrating resting mast cells was accompanied by an increase in the proportion of activated mast cells in tissues with high hypoxia scores. Roughly speaking, the proportions of infiltrating M0 macrophages experienced different degrees of increase in tissues with high hypoxia scores in all data sets, but the differences were significant only in GSE14520, GSE22058, GSE25097, GSE36376 and TCGA‐LIHC. In addition, the proportion of activated NK cells decreased in tissues with high hypoxia scores in almost all data sets but decreased significantly only in GSE10141, GSE14520 and GSE25097. These data suggest that hypoxia changes immune cell infiltration in the tumour tissues of HCC patients and that our 21‐gene hypoxia signature can be used to distinguish the difference in infiltrating immune cells between tissues with different hypoxia scores. Finally, we analysed the expression of programmed death‐1 (PD‐1)/programmed death‐ligand 1 (PD‐L1) and cytotoxic T‐lymphocyte antigen‐4 (CTLA‐4) in tissues with different hypoxia scores. In most data sets, there was no significant difference in PD‐L1 levels between tissues with high hypoxia scores and tissues with low hypoxia scores. However, in GSE22052, GSE25097, GSE9843 and TCGA‐LIHC, the expression of PD‐L1 was consistently significantly higher in tissues with high hypoxia scores than in tissues with low hypoxia scores (Figure 5D). The ranks of PD‐1 and CTLA‐4 levels in tissues with high hypoxia scores and low hypoxia scores were different in different data sets (Figure 5E,F). Interestingly, in multiple data sets, the ranks of PD‐1 levels in tissues with high hypoxia scores and low hypoxia scores were opposite to the ranks of CTLA‐4 levels in tissues with high hypoxia scores and low hypoxia scores. For example, in GSE22058, PD‐1 expression was higher in tissues with high hypoxia scores than in tissues with low hypoxia scores, but CTLA‐4 expression was lower in tissues with high hypoxia scores than in tissues with low hypoxia scores. These data suggest that the 21‐gene hypoxia signature may help to evaluate the response to immunotherapy in HCC patients.

## 4 DISCUSSION

Hypoxia can change the gene expression level in tumour cells. Therefore, the detection of the levels of specific genes can indirectly reflect hypoxia exposure in tumour cells or tissues.^24^ Although this method is not as accurate as the method using polarographic electrodes, it has high practicality. It can be predicted that with the development of high‐throughput detection technology and improvements in related algorithms, the accuracy of hypoxia assessments based on gene signatures will improve, and hypoxia gene signatures have great potential in research and clinical application. In the past decade, different hypoxia gene signatures have been reported, and some excellent signatures, such as Buffa's 15‐gene hypoxia signature, have proven to have the ability to indicate hypoxia in a variety of tumours.^9^ But few of these signatures were specially designed for HCC. An HCC‐suitable and tissue‐level hypoxia gene signatures still need to be developed.

In this study, we first performed a microarray analysis to obtain hypoxia‐responsive mRNAs profiles in three HCC cell lines and integrated results using the RRA algorithm. But not all these mRNAs are stable during hypoxia conditions. Part of hypoxia‐responsive mRNAs only changes after short‐term hypoxia exposure. With the prolongation of hypoxia, the level of these mRNAs may change. These mRNAs will not be used to assess the level of tissue hypoxia, because in cancer tissues, hypoxia is often long‐term and intermittent. Short‐term hypoxia *in* vitro experiments is different from long‐term intermittent hypoxia in tumour tissues.^25^ Intermediate and long‐term intermittent hypoxia in tissues in vivo has a pressure screening effect, which can promote a distinctive molecular profile that is different from that in vitro.^26^ Therefore, to obtain stable hypoxia‐induced mRNA that is not restricted by exposure time and applies to HCC tissues, we conducted qRT‐PCR verification on the integrated results. We found that the change of mRNAs of 21 genes after hypoxia exposure was stable and independent of the exposure model (acute, chronic and intermittent). We decided to use these 21 genes to construct our signature.

Most of the genes were reported that perform a hypoxia‐related biological function. CA9 has been frequently reported as a hypoxia marker that corrected PH changes caused by hypoxia by regulating hydrogen ions.^27^ The expression changes of HK2, PFKFB4 and GYS1 contributed to the hypoxia‐induced disturbance of carbohydrate metabolism.^28^ EGLN3 is induced by hypoxia and influences the stability of HIF1A.^29^ HILPDA is associated with lipid droplet formation under hypoxia.^30^ BNIP3 and BNIP3L mediate hypoxia‐induced autophagy which is a survival mechanism that promotes tumour progression including HCC.^31^, ^32^ For other genes in the signature, the results of qRT‐PCR confirmed that they are indeed hypoxia‐responsive. But the specific function has not been reported yet and needs to be clarified in future research.

Before further analysis, we performed cell‐level validation in 3 other public data sets, demonstrating that our 21‐gene hypoxia signature can indicate hypoxia exposure. At the tissue level, we found that our 21‐gene hypoxia signature showed a good correlation with the 6 signatures reported (highly cited) by previous studies. Therefore, we believe that the novel 21‐gene hypoxia signature has excellent robustness in the assessment of hypoxia exposure. After calculating hypoxia scores using the 21‐gene hypoxia signature, we found that the hypoxia scores in the tumour tissues of HCC patients in 11 data sets were obviously grouped into 2 clusters, indicating that hypoxia exposure in HCC patients is not the same. In other words, some patients may respond to anti‐hypoxia treatment while other patients do not need anti‐hypoxia treatment because there is no excessive exposure to hypoxia. Currently, the efficacy of anti‐hypoxia treatment is not satisfactory. Therefore, screening patients using a hypoxia signature before formulating a treatment program may change this situation.

In recent years, the molecular classification of HCC has received attention. A series of molecular classification strategies were unveiled based on the difference in the multi‐omics profiles of HCC. For example, Chiang reported a CTNNB + Proliferation + Interferon + Poly7 classification in HCC based on 91 oncogenes.^33^ Shimada et al provided a molecular classification based on the immunological characterization of HCC.^34^ By analysing the Metabolism‐associated, Young et al provided a metabolism‐associated molecular classification system.^35^ The molecular classifications provide important clues to explain the developmental mechanism and personalized treatment of HCC. We believe that hypoxia‐associated molecular classification could be also established by considering hypoxia's significant role in HCC. We used the 21‐gene hypoxia signature to perform subtype analysis on HCC patients. In 2 independent cohorts (TCGA and GSE14520), the 21‐gene hypoxia signature effectively classified patients into subtypes. The obtained subtypes differed regarding clinical characteristics, including AFP level, TNM stage and prognosis. Therefore, from the perspective of precision medicine, the classification of HCC based on hypoxia exposure has clinical implications for determining prognosis and developing personalized treatment plans, which may benefit specific patient groups.

Except for the molecular classifications, the 21‐gene hypoxia signature could be used to calculate the hypoxia score in HCC tissues. We found that the hypoxia score of HCC tissues associated with other clinical features such as AFP level, TNM stage, BCLC stage, CLIP stage, vascular invasion and metastasis. A high hypoxia score calculated by 21‐gene signature was suggested as an independent risk factor for survival. The hypoxia score system could effectively estimate the survival of patients without dependence on TNM staging. Meanwhile, a Lasso‐cox model was constructed using hypoxia scores and the TNM stage as indicators. The model can well predict the OS rate of HCC patients and can indicate the recurrence of HCC. These results were validated in multiple cohorts, suggesting the value of the 21‐gene hypoxia signature in clinical application. The multiple data sets used for validation include both RNA sequencing data and microarray data. Hence, the 21‐gene hypoxia signature may have a cross‐platform feature that indicated a great application potential.

Indicating or predicting the prognosis was not the only application of the 21‐gene hypoxia signature. Using our signature, we were also able to map out the hypoxia caused molecule landscape. With the development and popularization of high‐throughput technology, especially The Cancer Genome Atlas (TCGA) project, which has been gradually completed, increasingly betterwell‐organized genomic, epigenomic, transcriptomic and proteomic data have become available. This provides an unprecedented opportunity for an in‐depth exploration of the roles and mechanisms of hypoxia in vivo tissues. Some recent studies have attempted to use these signatures to reveal molecular changes in tumour cells caused by hypoxia at multi‐omic levels. For example, the pan‐cancer study by Bhandari et al showed molecular landmarks of tumour hypoxia across cancer types.^19^ The integrative study by Ye et al suggested that hypoxia‐associated molecular features are closely related to the drugs used for tumour treatment.^20^ However, the signatures used in the aforementioned studies are not HCC‐suitable, and because they are pan‐cancer studies, the coverage and depth of the research content for HCC are limited. Therefore, we believe that using the 21‐gene signature to specifically depict full‐scale hypoxia‐related molecular landscapes in HCC from a multi‐omics perspective will help update knowledge and finally benefit HCC patients. As shown in Figure 1, in our ongoing study we are trying to use the 21‐gene hypoxia signature to describe hypoxia‐related molecular landscapes from genomic, epigenomic, transcriptomic and proteomic perspectives in HCC tissues. The purposes of this study were to provide tissue‐level evidence for explaining the mechanism of hypoxia in HCC, to provide a comprehensive overview of the role of hypoxia in HCC, and to find the possible treatment or diagnostic targets. We have already collected preliminary data that indicated transcriptomic (mRNA, microRNA and lncRNA), genomic (copy number variation), epigenomic (methylation and alternative splicing of RNA), and proteomic alterations between patients with high hypoxia score and low hypoxia score. We hope that in the future our complete data can inspire and help other researchers, improve research efficiency, and narrow the scope of researches.

In the last part of this study, the effect of hypoxia on immune cell infiltration patterns in HCC tissues was investigated. It was found that hypoxia‐induced the infiltration of immune cells in HCC tissues and that the proportion of specific types of immune cells changed. The current evidence is inadequate to clarify the role and significance of immune cells with changes in their proportions. However, based on existing reports, they might be involved in hypoxia‐induced immune escape. Hypoxia may affect the expression levels of immune checkpoint regulators, such as PD‐1 and PD‐L1, suggesting that hypoxia may be related to the efficacy of immunotherapy.

In summary, the 21‐gene signature developed in this study can effectively estimate hypoxia exposure in HCC tissues. The molecular classification derived from the signature indicated clinical characteristics and prognosis in HCC. Hypoxia score calculated by the 21‐gene signature is an independent risk factor of HCC patients and related other clinical characteristics. The predictive model that is available to effectively predict the outcome of HCC patients by appropriately stratifying the hypoxia score. In the personalized treatment of HCC patients, the assessment of the degree of hypoxia is strongly recommended to benefit specific patient groups.

## CONFLICT OF INTEREST

The authors declare no potential conflicts of interest.

## AUTHOR CONTRIBUTIONS

**Qiangnu Zhang:** Data curation (lead); Formal analysis (lead); Investigation (lead); Methodology (lead); Visualization (lead); Writing‐original draft (lead). **Lijun Qiao:** Conceptualization (equal); Funding acquisition (equal); Investigation (equal); Methodology (equal); Writing‐original draft (equal). **Juan Liao:** Funding acquisition (equal); Validation (equal); Visualization (equal); Writing‐review & editing (equal). **Quan Liu:** Methodology (equal); Visualization (equal); Writing‐review & editing (equal). **Pengyu Liu:** Data curation (equal); Investigation (equal); Writing‐original draft (equal). **Liping Liu:** Conceptualization (equal); Funding acquisition (equal); Project administration (equal); Supervision (lead); Writing‐review & editing (equal).

## Supporting information

Fig S1‐S3

Table S1‐S5

## Data Availability

The data sets supporting the conclusions of this article are available in the TCGA data portal (https://portal.gdc.cancer.gov/) and the Gene Expression Omnibus (GEO, https://www.ncbi.nlm.nih.gov/geo/).
