# Supplementary material for: A novel hypoxia gene signature indicates prognosis and immune microenvironments characters in patients with hepatocellular carcinoma
Source: J Cell Mol Med. 2021 Feb 22;25(8):3772–84. doi: 10.1111/jcmm.16249 (PMC8051726; doi:10.1111/jcmm.16249)
Supplement: Supplementary file 1 — Fig S1‐S3 [file JCMM-25-3772-s001.docx]

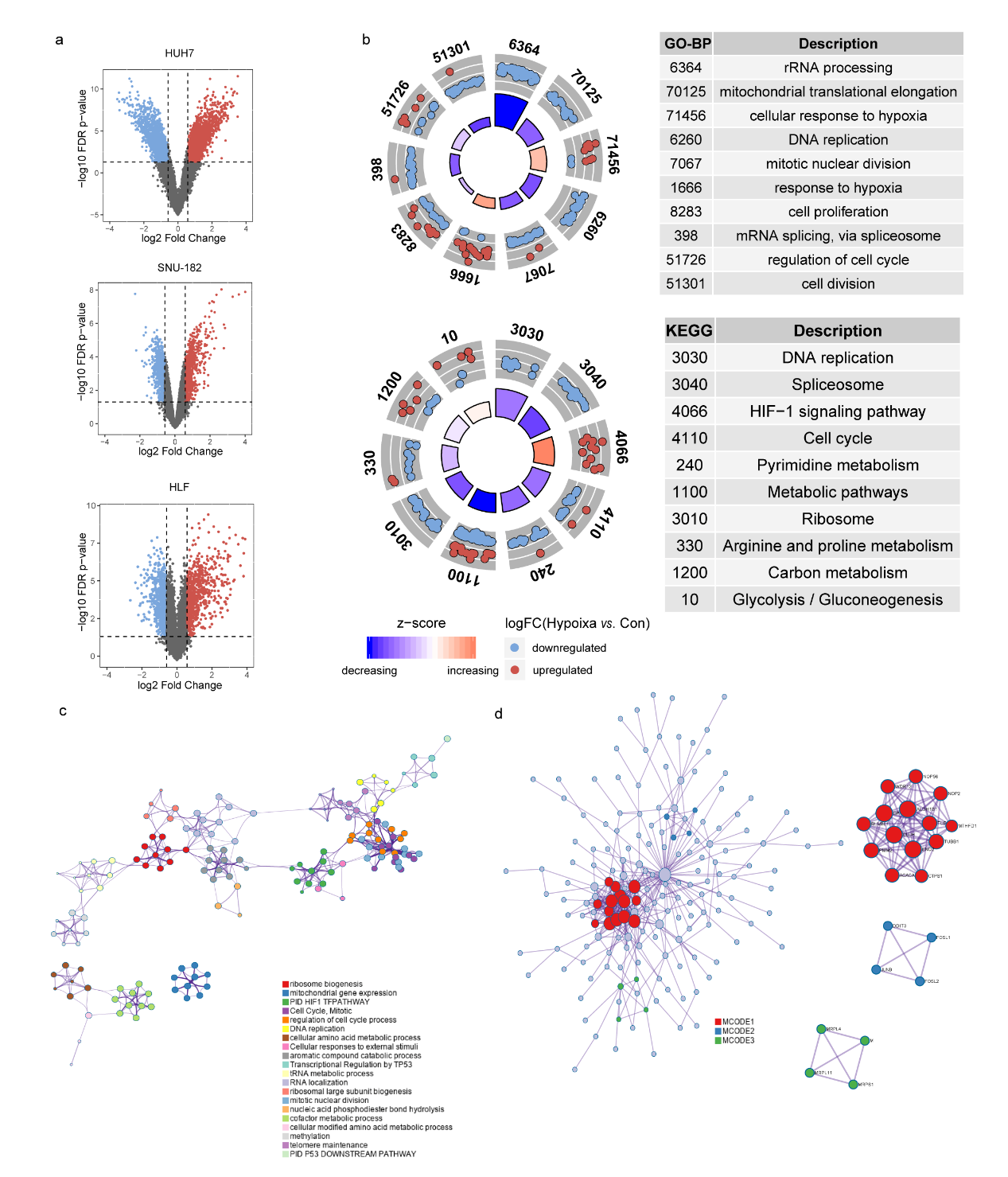


Supplemental Figure 1. The mRNA changes and functional analysis of hypoxia-treated HUH7, SNU-182, and HLF cells. a) Distribution of differentially expressed mRNAs in HUH7, SNU-182, and HLF cells after 24-h hypoxia exposure. b) Top 10 (sorted according to the *P* value) enrichment analysis results for GO biological processes and Kyoto Encyclopedia of Genes and Genomes (KEGG) pathways using the integrated intersection of differentially expressed mRNAs in HUH7, SNU-182 and HLF cells after 24-h hypoxia exposure. c) The terms in the functional enrichment analysis form a network. d) The protein products of the integrated differentially expressed mRNAs constitute the protein-protein interaction enrichment network.


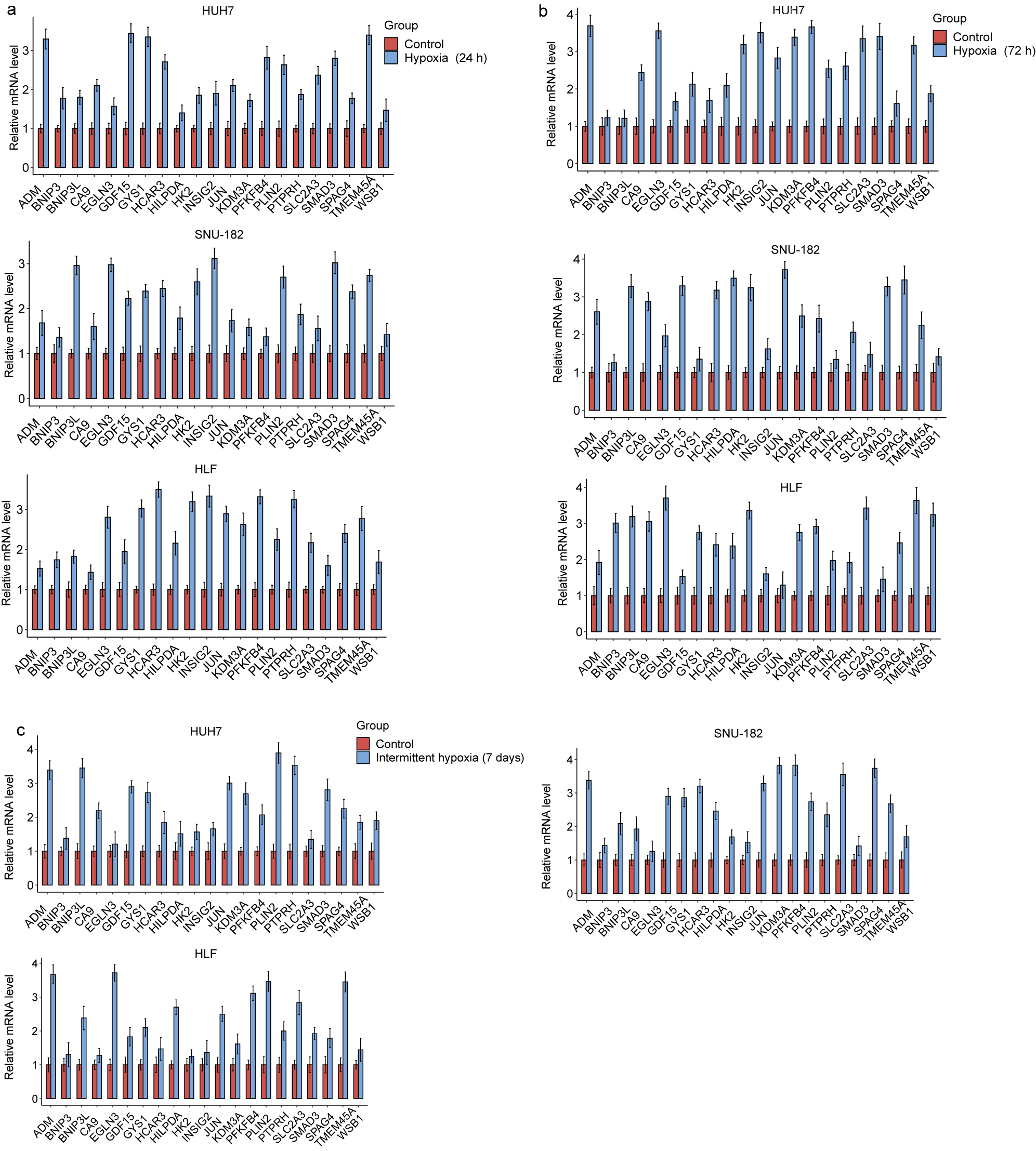


Supplemental Figure 2. qRT-PCR validation for 21 of stable hypoxia-responsive mRNAS. a) mRNA changes of 21 candidate genes in HUH7, SNU-182 and HLF cells at 24 h.b) mRNA changes of 21 candidate genes in HUH7, SNU-182 and HLF cells at 72 h.c) mRNA changes of 21 candidate genes in HUH7, SNU-182 and HLF during one week intermittent hypoxia. All *P*<0.05 compared with control.


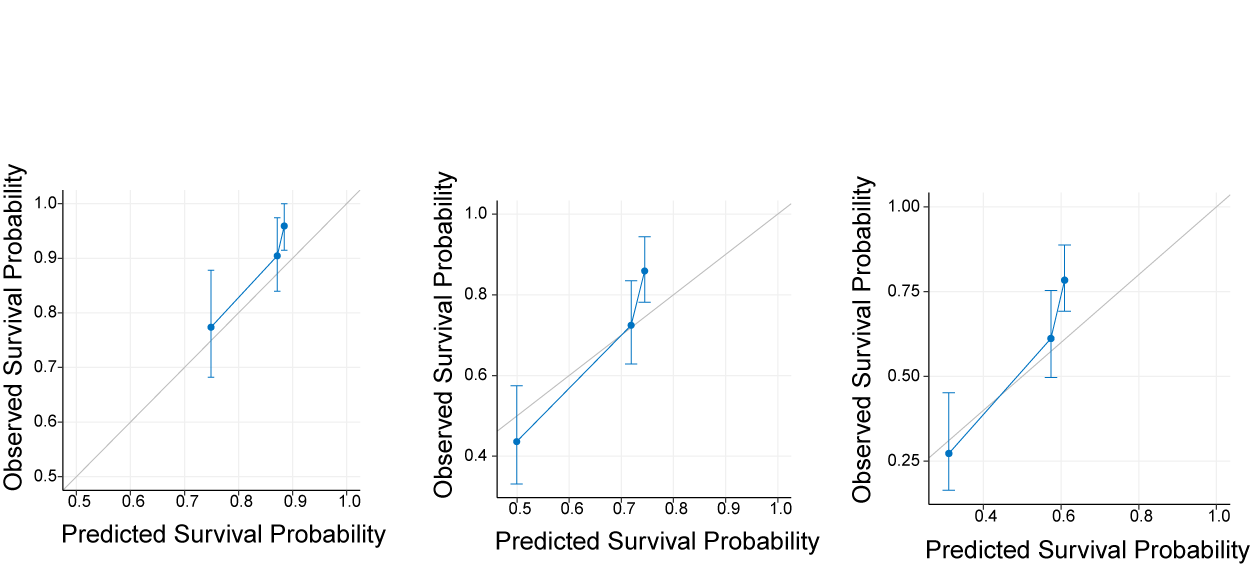


Supplemental Figure 3. Calibration curve for the LASSO-Cox regression model for the prediction of 1-, 3- or 5-year overall survival in GSE14520. This LASSO-Cox regression model uses the hypoxia score and TNM stage as factors and is based on TCGA-LIHC data.
